# A Hospital-Based Study of Vitamin D Levels in Children With Recurrent Respiratory Infections

**DOI:** 10.7759/cureus.27864

**Published:** 2022-08-10

**Authors:** Amol P Jaybhaye, Avinash L Sangle, Deepak Ugra, Ravindra Y Chittal

**Affiliations:** 1 Paediatrics, Consulting Paediatrician, Aurangabad, IND; 2 Department of Paediatrics, Mahatma Gandhi Mission Medical College, Aurangabad, IND; 3 Paediatrics, Lilavati Hospital and Research Centre, Mumbai, IND

**Keywords:** vitamin d deficiency, nonallergic rhinitis, acute otitis media, mucosal immunity, respiratory tract infections

## Abstract

Background

The association of sub-normal vitamin D levels with respiratory tract infections in children has been a topic of interest in the recent literature. Vitamin D insufficiency has been explored as a modifiable risk factor in the management of pediatric recurrent respiratory tract infections.

Methodology

This hospital-based study included 108 children as cases aged six months to 15 years who were enrolled either as inpatients or outpatients with recurrent respiratory infections. In total, 55 healthy children of the same age group attending the hospital for vaccination and routine check-ups during the study period were included as controls. Venous blood specimens were collected from cases and controls to study serum 25-hydroxyvitamin D.

Results

The mean age of the cases and controls was 68.25 ± 40.3 months and 52.6 ± 40.9 months, respectively. Among the cases, 25% were vitamin D deficient and 75% had vitamin D insufficiency. The difference in proportions of vitamin D sufficiency status among cases and controls was statistically significant (p < 0.001).

Conclusions

There was a very high prevalence of vitamin D deficiency among children with recurrent respiratory infections compared to controls. The vitamin D status assessment should be included in the management of children with recurrent respiratory infections.

## Introduction

Respiratory tract infections in children are commonly encountered in clinical practice. These infections are one of the most frequent reasons for consulting a physician and contribute significantly to childhood morbidity and mortality. Globally, deaths across all age groups due to respiratory tract infections were reported to be 2.65 million in 2013 [1-4]. India, Bangladesh, Indonesia, and Nepal accounted for 40% of the global acute respiratory infection mortality [5]. Reports suggest that children under five years of age suffer about five episodes of acute respiratory infection per year. Moreover, acute respiratory infections are a leading cause of deafness as sequelae of acute otitis media [5]. There is a need to identify the modifiable factors that can impact the prevalence and management of respiratory infections among children. Vitamin D deficiency modulates the immune mechanisms associated with the risk of respiratory infections during childhood [2]. Vitamin D levels are significantly lower among children with recurrent respiratory tract infections. Studies have suggested further exploring the relationship between vitamin D deficiency and respiratory infections in children [2,6,7]. With this perspective, this hospital-based study was undertaken to evaluate the vitamin D levels among children presenting with recurrent respiratory infections at a tertiary care center in Mumbai, India.

## Materials and methods

This hospital-based, observational study included 108 children (cases) between the ages of six months to 15 years who were admitted as inpatients or seen in the outpatient department of the tertiary care hospital with recurrent respiratory infections. The Ethics Committee of Lilavati Hospital and Research Centre, Mumbai, approved the study protocol (IEC/LHRC/01/12, dated August 14, 2012). The study duration was from September 2012 to April 2013. The inclusion criteria for the cases were: (i) ≥six respiratory infections per annum; (ii) ≥one respiratory infection involving the upper airways from September to April; (iii) ≥three respiratory infections per year involving the lower airways; (iv) otitis media, three episodes within six months or four episodes within 12 months; (v) recurrent infectious rhinitis, that is, more than five episodes per year; or (vi) recurrent pharyngitis or tonsillitis, that is, more than three episodes within 12 months [8].

Children with respiratory allergies (including asthma), congenital heart diseases, gastroesophageal reflux disease, immunodeficiency disorders, and congenital structural anomalies were excluded from the study. In total, 55 healthy children aged six months to 15 years seen in the outpatient department of the same hospital for vaccination and routine check-ups during the study period were included as controls. Informed consent was obtained from the parents or the guardians of the cases and controls. Venous blood specimens were collected from cases and controls to study serum 25-hydroxy vitamin D (25(OH)D). The blood samples were collected when cases did not have any acute respiratory infection. The Roche Elecsys Vitamin D3 (25OH) analyzer using the electrochemiluminescence immune assay (ECLIA) method was used to measure 25(OH)D. The lower detection limit of this method was 3 ng/mL. The reference range for serum vitamin D levels is as follows: vitamin D deficiency: less than 20 ng/mL, vitamin D insufficiency: 21-29 ng/mL, and vitamin D sufficiency: 30-100 ng/mL [9]. The chi-square test was used to analyze the proportions using GraphPad QuickCalcs. A p-value of <0.05 was considered statistically significant.

## Results

The mean age of the cases and controls was 68.25 ± 40.3 months and 52.6 ± 40.9 months, respectively. In total, 38 children were 6-60 months of age, 13 children were 61-120 months of age, and four children were >120 months (Table 1).

**Table 1:** Age group distribution between cases and controls.

Age (months)	Group		Total
	Cases	Controls	
60 or less	57 (52.8%)	38 (69.1%)	95 (58.3%)
61–120	40 (37%)	13 (23.6%)	53 (32.5%)
>120	11 (10.2%)	4 (7.3%)	15 (9.2%)
Total	108 (100%)	55 (100%)	163 (100%)

The cases and control groups were not significantly different with respect to sex (approximately 57% males and 43% females in both groups).

**Figure 1:**
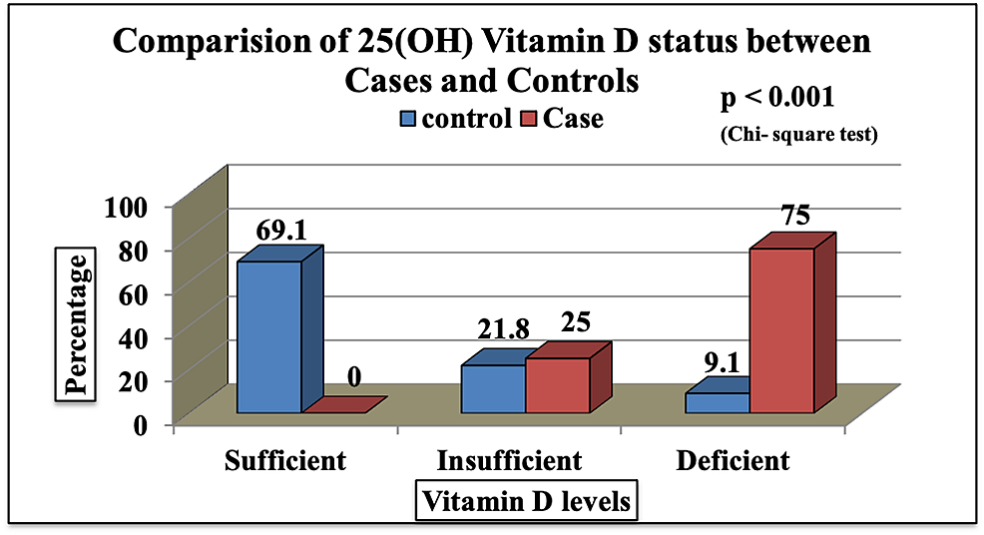
Vitamin D status of patients.

Figure 1 shows that there was no child with vitamin D sufficiency among the cases with recurrent respiratory tract infections. One-fourth of the cases had vitamin D insufficiency and three-fourths of the cases had vitamin D deficiency. These differences in proportions of vitamin D sufficiency status among cases and controls were statistically significant with a p-value of less than 0.001.

Table 2 shows the number of cases and controls with vitamin D sufficiency status along with the comparison of 25(OH)D between cases and controls with each inclusion criteria.

**Table 2:** Comparison of 25(OH)D between cases and controls with each inclusion criteria.

Group	25-hydroxy vitamin D			Total
	Sufficient	Insufficient	Deficient	
Control	38 (69.1%)	12 (21.8%)	5 (9.1%)	55
Case	0 (0%)	27 (25%)	81 (75%)	108
Criteria 1 (≥6 respiratory infections per annum)	0 (0%)	13 (29.5%)	31 (70.5 %)	44
Criteria 2 (≥ respiratory infection/s per month involving the upper airways from September to April)	0 (0%)	8 (22.9%)	27 (77.1%)	35
Criteria 3 (≥3 respiratory infections per annum involving lower airways)	0 (0%)	1 (14.3%)	6 (85.7%)	7
Criteria 4 (otitis media 3 episodes within 6 months/4 episodes within 12 months)	0 (0 %)	1 (33.3%)	2 (66.6%)	3
Criteria 5 (recurrent infectious rhinitis >5 episodes per year)	0 (0 %)	3 (21.4%)	11 (78.6%)	14
Criteria 6 (recurrent pharyngitis or tonsillitis, i.e., more than three episodes within 12 months)	0 (0 %)	1 (20%)	4 (80%)	5

## Discussion

The study results reflect the higher proportion of vitamin D insufficiency among the cases of recurrent respiratory tract infection in children. Similar to our findings, Wayse et al. (2004) reported the role of subclinical deficiency of vitamin D as a significant risk factor for lower respiratory tract infections in Indian children under five years of age [10]. Özdemir et al. (2016) reported that mean vitamin D levels in children with recurrent respiratory infections was 11.97 ± 4.04 ng/mL, in children with chronic cough was 13.76 ± 4.81 ng/mL, and in the control group was 31.91 ± 18.79 ng/mL, with a statistically significant difference between study groups. Thus, vitamin D deficiency in children was associated with an increased frequency of recurrent respiratory infections and chronic cough [11]. A recent study from the Telangana state of India has reported the association of vitamin D levels with recurrent respiratory infections [12]. Zhang et al. (2015-2018) also reported a correlation between vitamin D deficiency and recurrent respiratory infections in China [13]. Studies have explored the underlying mechanism for the relation of vitamin D with respiratory infections. It has been suggested that vitamin D has a role in immunity, and insulin-like growth factor 1 promotes the synthesis and secretion of immunoglobulins. Additionally, vitamin D can facilitate the proliferation and differentiation of T and B lymphocytes. Studies have also reported that CD4+ and CD3+ levels were lower in the vitamin D-deficient group, which was amenable to vitamin D supplementation. Moreover, immunoglobulin (Ig)A, IgM, and IgG levels were increased after treatment with vitamin D supplements. Vitamin D was found to improve humoral immunity and decrease the incidence of respiratory tract infections [14]. Vitamin D deficiency is considered to induce weakness of the muscles, especially the diaphragm and intercostals. This likely makes it difficult to clear the secretions of the respiratory tract and facilitate infections [15]. Esposito and Lelii reviewed the literature on vitamin D and respiratory infections and found that vitamin D deficiency is a risk factor for tuberculosis, bronchiolitis, and recurrent otitis media in children. They concluded that maintaining adequate vitamin D levels could be a low-cost and effective method to prevent some respiratory tract infections [2]. Literature from various parts of the world supports the role of vitamin D deficiency in respiratory infections [16-18].

The study limitations include a hospital-based small sample data with a study design that cannot provide a cause-effect relationship. More extensive studies with robust design are needed on the subject for a better understanding.

## Conclusions

Children with recurrent respiratory tract infections exhibited a significantly higher proportion of vitamin D-deficient subjects. Vitamin D levels should be evaluated in children with recurrent respiratory tract infections and treated timely for better management of the problem.
